# Fourier analysis using the number of COVID-19 daily deaths in the US

**DOI:** 10.1017/S0950268821000522

**Published:** 2021-03-04

**Authors:** Yoshiyasu Takefuji

**Affiliations:** Faculty of Environment and Information Studies, Keio University, 5322 Endo, Fujisawa 252-0882, Japan

**Keywords:** COVID-19, cycle length, Fourier analysis, resurgence

## Abstract

Fourier analysis can provide policymakers useful information for analysing the pandemic behaviours. This paper proposes a Fourier analysis approach for examining the cycle length and the power spectrum of the pandemic by converting the number of deaths due to coronavirus disease 2019 in the US to the frequency domain. Policymakers can control the pandemic by using observed cycle length whether they should strengthen their policy or not. The proposed Fourier method is useful for analysing waves in other medical applications.

## Introduction

Fourier power spectrum analysis technology has been used in many medical applications [1–4]. This paper shows how to use the traditional Fourier power spectrum analysis for examining the period of the pandemic wave cycle. The number of daily deaths due to coronavirus disease 2019 (COVID-19) can be simply used for grading the success of individual policies in many nations. The number of daily deaths is converted to the frequency domain in order to observe the behaviour of the pandemic. Instead of observing the number of deaths directly, the converted peak spectrum can simply indicate the strength and the tendency of the pandemic waves. The frequency domain expression allows policymakers to understand the cycle length of the pandemic whether they should strengthen the policy or not.

Data on the number of daily deaths in the US due to COVID-19 from 31 December 2019 to 24 October 2020 is downloadable (JHU 2020). Fast Fourier transform (FFT) technology is normally used for real-time analysis. However, this paper uses the modified static data, d.csv from 1 March to 24 October since the first death in the US was reported on 1 March 2020 [5].

The COVID-19 pandemic of 2020 is one of the largest pandemics ever recorded, along with the Spanish influenza pandemic of 100 years ago and the AIDS pandemic of the 1990s. Data analysis is playing an increasingly important role in non-pharmaceutical epidemic control in the early stages of an epidemic, since we have several vaccines but not available yet in public and no effective therapy against COVID-19.

This study proposes to perform Fourier analysis on the time series of COVID-19 death tolls to provide useful information to policy makers, and provides a programme that can be implemented in practice. In particular, it is very interesting that Fourier analysis can be used to analyse epidemic wave breaks, which will be useful information for policy makers.

## Methods

In many cases, the number of infected people using polymerase chain reaction tests or other methods is used as a statistic for monitoring COVID-19 epidemics, but there is criticism that the detection accuracy of such tests is very poor [6]. In addition, since unaware infected persons and latently infected persons are not tested, monitoring by the number of infected persons is associated with uncertainty in understanding the full extent of the COVID-19 epidemic unless massive testing such as Wang [7] is conducted.

However, since the number of deaths associated with COVID-19 is known to have occurred after hospitalisation, treatment and isolation due to the effects of COVID-19, the possibility of false negatives is reduced, although the detection accuracy of the test is affected, and many cases of unconscious or latent infections are eliminated. For this reason, it is relatively better to use the number of deaths, as shown in this paper, rather than the number of infected people as a statistic for monitoring an epidemic.

**Fig. 1.**
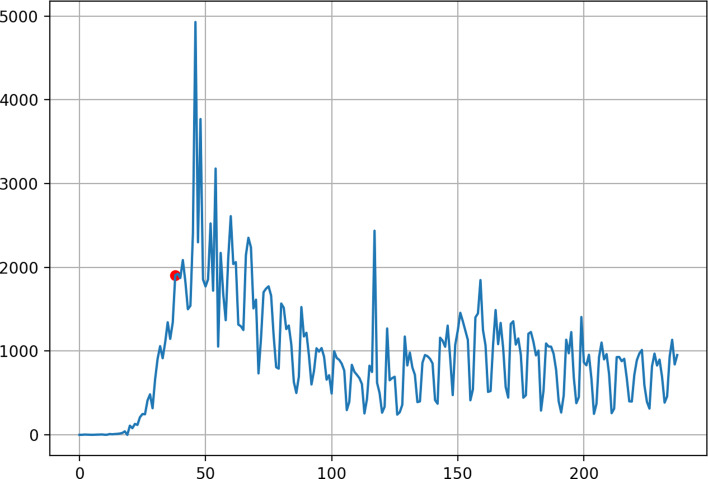
Number of daily deaths in the US from 1 March 2019 to 24 October 2020. X-axis: days, Y-axis: the number of daily deaths, red point indicating 8 April.

In the FFT analysis, the number of samples or the total number of 238 days is used in this paper. Figure 1 shows the number of daily deaths in the US where ‘0’ on the X-axis represents 1 March 2020, while ‘237’ on the X-axis represents 24 October 2020. The red point indicates 8 April in Figure 1. The graph in Figure 1 can be generated by us-fft0.py: https://github.com/ytakefuji/fourier/us-fft0.py

To calculate the wave cycle or period, the analysed data is split into two data groups by the splitting day.

**Fig. 2.**
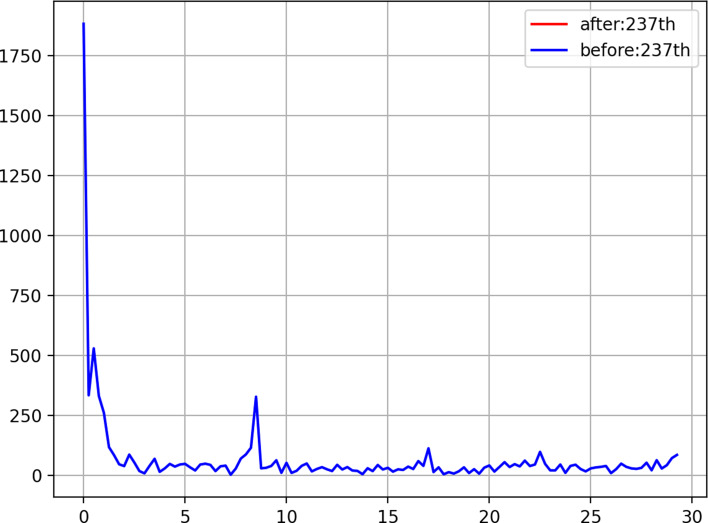
Result of spectrum analysis using FFT. X-axis: the cycle of days, Y-axis: the power spectrum.

Figure 2 shows the result of spectrum analysis using 200 days from 8 April to 24 October 2020. Figure 2 depicts that the peak power spectrum is the 7-day cycle of the pandemic. Since, the US death reporting is based on the cycle of 7 days a week [5]. The graph of Figure 2 can be generated by us-fft2.py by using 237 days. The program source of us-fft2.py is available at the following site: https://github.com/ytakefuji/fourier/us-fft2.py

If we use a bandpass filter with the specified centred frequency, we can remove the 7-day cycle spectrum from spectrums. For example, the 7-day cycle can be completely eliminated by the bandpass filter with 7-day centred frequency.

However, the bandpass filter may harm the final spectrum analysis result.

Figure 2 shows the power spectrum where the peak spectrum shows the cycle length by using 200 days data with splitting data on 8 April where the peak spectrum is the 7-day cycle. Note that in the graph we can ignore the spectrum below the 1-day cycle on the X-axis. Data of 238 days on the number of daily deaths from 1 March 2019 to 24 October 2020 are split into two groups by the splitting day.

Dataset of 238 days on the number of daily deaths in the US, d.csv file is available at the following site: https://github.com/ytakefuji/fourier/blob/main/d.csv

**Fig. 3.**
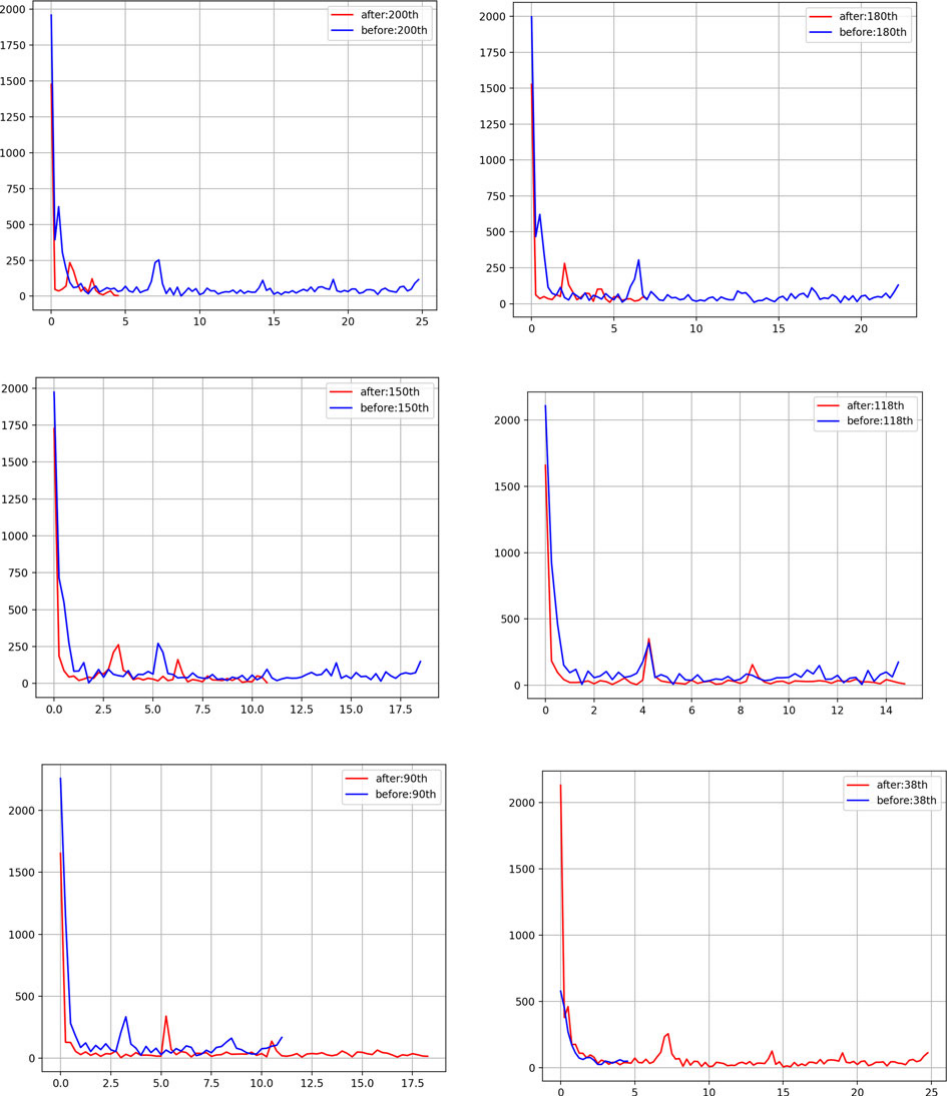
(a) Spectrum using interval [200:238] days and [0:200] days split on 8 April, (b) spectrum using interval [180:238] days and [0:180] days split on 28 April, (c) spectrum using interval [150:238] days and [0:150] days split on 28 May, (d) spectrum using interval [118:238] days and [0:118] days split on 29 June, (e) spectrum using interval [90:238] days and [0:90] days split on 27 July, (f) spectrum using interval [38:238] days and [0:38] days split on 17 September.

Figure 3a–f shows the calculated spectrum using 238 days with altering the splitting days of 8 April, 28 April, 28 May, 29 June, 27 July and 17 September, respectively. The graphs in Figure 3a–f can be generated by us-fft2.py with changing the number of days as ‘day’ parameter.

In the us-fft2.py program, the splitting ‘day’ indicates the interval [day:238] in red line and the interval [0: day] in blue line, respectively. For example, note that interval [180:238] means the day range of 180−237 in the Python program. [0: day] is equivalent to the day range of 0 to (day-1) in the Python program.

Figure 3a shows the spectrum using interval [200:238] days and [0:200] days split on 8 April, in Figure 3b spectrum using interval [180:238] days and [0:180] days split on 28 April, in Figure 3c spectrum using interval [150:238] days and [0:150] days split on 28 May, in Figure 3d spectrum using interval [118:238] days and [0:118] days split on 29 June, in Figure 3e spectrum using interval [90:238] days and [0:90] days split on 27 July 27 and in Figure 3f spectrum using interval [38:238] days and [0:38] days split on 17 September, respectively.

Figure 3a–f indicates that the shorter cycle length describes the resurgence of the pandemic while the longer cycle suppressing the pandemic. The tendency of the pandemic can be clearly described by the cycle length using the power peak spectrum. By converting the number of daily deaths data to the spectrum frequency domain with the FFT technology, the pandemic behaviour or the cycle length can be simply observed by the novice. Figure 3d shows that the peak of power spectrum of days before 29 June is the same as that of after 29 June. This induces that 29 June is the day of the first wave end in the US pandemic. The shorter cycle length indicates that the policy should be strengthened for mitigating the pandemic while the longer cycle length allows us to relax the policy.

## Conclusion

The FFT technology allows us to observe the behaviour of the pandemic not by the number of daily deaths but by the cycle length of the power peak spectrum in the frequency domain. Policymakers can control their policies based on the cycle length in the pandemic waves. The proposed Fourier method is useful for analysing wave behaviours in other medical applications.

This paper has not been published and is not under consideration for publication elsewhere. The author has no conflict of interest. This research has no fund. The author has read the paper and has approved its submission.

Data and source programs are available:

https://github.com/ytakefuji/fourier/blob/main/d.csv

https://github.com/ytakefuji/fourier/us-fft0.py

https://github.com/ytakefuji/fourier/us-fft2.py
